# Role of Renal Oxygenation and Mitochondrial Function in the Pathophysiology of Acute Kidney Injury

**DOI:** 10.1159/000363545

**Published:** 2014-09-24

**Authors:** Noureddin Nourbakhsh, Prabhleen Singh

**Affiliations:** Division of Nephrology-Hypertension, University of California San Diego School of Medicine, and VA San Diego Healthcare System, San Diego, Calif., USA

**Keywords:** Oxygenation, Mitochondrial function, Metabolism, Transport

## Abstract

There are unique features of renal oxygenation that render the kidney susceptible to oxygen demand-supply mismatch and hypoxia. Renal oxygen consumption by oxidative metabolism is closely coupled to and driven by tubular transport, which is linked to the filtered solute load and glomerular filtration rate (GFR). In turn, filtered solute load and GFR are dependent on the renal blood flow. Hence, changes in renal blood flow increase oxygen delivery but also increase oxygen demand (consumption) simultaneously by increasing the tubular workload of solute transport. The renal blood flow to different regions of kidney is also inhomogeneous, increasing the oxygen demand-supply mismatch in particular areas such as the outer medulla which become more susceptible to injury. Thus, tubular transport and oxidative metabolism by mi ochondria are closely coupled in the kidney and are the principal determinants of intrarenal oxygenation. Here we review the published literature characterizing renal oxygenation and mitochondrial function in ischemic and sepsis-associated acute kidney injury (AKI). However, the coupling of transport and metabolism in AKI has not been examined. This is a potentially fruitful area of research that should become increasingly active given the emerging data linking renal oxygenation and hypoxia to acute and chronic dysfunction in the kidney.

## Renal Oxygenation and Mitochondrial Function under Physiological Conditions

For optimal function of intracellular organelles, cells require continuous ATP generation. Both oxidative and glycolytic pathways produce ATP, but oxidative phosphorylation is more efficient (38 molecules of ATP per mole of glucose vs. 2 molecules of ATP with glycolysis). Hence, most cells have an incessant need for oxygen to generate ATP via mitochondrial oxidative phosphorylation. While a lack of oxygen supply is a definite cellular stress, excess oxygen can also lead to oxidant damage. Hence, tight regulation of tissue oxygenation to prevent hypoxia or hyperoxia is critical for survival.

Determinants of renal oxygenation and tissue oxygen tension (PO_2_) include renal blood flow (RBF) and the oxygen content of arterial blood, the oxygen consumed by the cells, and arterial-to-venous oxygen shunting, which describes the diffusion of oxygen from preglomerular arteries to postglomerular veins without oxygen being available to the cell for consumption [1]. The kidney enjoys a high blood flow (nearly 25% of the cardiac output), which is needed to sustain GFR. Compared to other major body organs, the renal oxygen consumption (QO_2_) per gram of tissue is high, second only to that of the heart (2.7 vs. 4.3 mmol/kg/min for the heart) [2]. This is largely driven by the high RBF since the renal oxygen extraction is low. It has been hypothesized that renal arterial-to-venous oxygen shunting is an adaptation to prevent hyperoxia due to the high renal perfusion needed to sustain the GFR, but this same adaptation can be detrimental in conditions of oxygen demand-supply mismatch [1].

There are some unique features of renal oxygenation that render the kidney susceptible to hypoxia. Nearly 80% of the oxygen consumed for ATP production supports active sodium transport (TNa), while the basal metabolic rate accounts for only 15–20% of the total QO_2_ [3]. A linear relationship between TNa and QO_2_ has been repeatedly demonstrated, suggesting that a close coupling exists between transport and metabolism in the kidney [4]. Hence, renal oxygenation is intimately related to the GFR and tubular reabsorption. Because TNa accounts for most of the oxygen consumption in the kidney, the renal QO_2_ varies with the GFR. This brings us to another unique feature of renal oxygenation: unlike other organs where an increase in blood flow uniformly improves the net oxygen status by increasing the oxygen supply, an increase in RBF increases the GFR and the filtered reabsorptive load and consequently the QO_2_. Thus, in the kidney, an increase in oxygen supply also increases the oxygen demand simultaneously, unless accompanied by a reduction in transport.

Another peculiarity of the kidney is its inhomogeneous blood supply. The cortex receives nearly 20% of the cardiac output, with a tissue PO_2_ of 50–60 mm Hg, while the blood supply to the medulla is limited to just 5–10% of the total RBF, with a tissue PO_2_ of 10–20 mm Hg [5]. The low blood flow is necessary to preserve medullary osmotic gradients for urinary concentration but leaves the medullary tissue at the brink of hypoxia, especially the outer stripe of outer medulla where the S3 segment of the proximal tubule and medullary thick ascending limbs (TAL) lie. These are also the segments most vulnerable to injury. The high metabolic requirements of the medullary TAL also contribute to the low tissue PO_2_ in this region. The oxygen costs of Na reabsorption increase along the nephron, with the proximal tubule being most metabolically efficient. Although the bulk of Na transport occurs in the proximal tubule, only 60% of this is active transport. The medullary TAL are the site of significant active Na transport needed to generate the osmotic gradients and they have a higher NaKATPase activity than the proximal tubules. The metabolic energy used per milligram of protein is 50% higher in the TAL than in proximal tubules, and it is further increased in the distal tubule [4]. An increase in Na transport in the TAL without an increase in oxygen supply can aggravate medullary hypoxia. Brezis and colleagues [5] clearly demonstrated that inhibition of Na transport in the TAL and proximal tubule by specific diuretics increased PO_2_ in the medulla and cortex, respectively. Hence, tubular transport and the related QO_2_ are the principal determinants of intrarenal oxygenation.

The kidney derives most of its energy from oxidative metabolism (95–99%), and both proximal tubules and TAL are rich in mitochondria [4]. In addition to ATP generation by oxidative metabolism, mitochondria play a significant role in the generation and detoxification of reactive oxygen species, cellular calcium homeostasis, cell survival and death (autophagy and apoptosis), and other cell signaling pathways [6]. They are dynamic organelles able to respond to the changing energy demands of the cells, and they play a key role in the adaptation to cellular stress in highly metabolic organs. Mitochondrial dysfunction has been actively pursued and strongly implicated in the pathophysiology of highly metabolic organs such as the heart, brain, and liver and more recently the kidney.

Mitochondrial morphology plays an important role in cellular physiology and pathophysiology and is regulated by frequent fission and fusion events [7]. Fission is the division of mitochondria within a cell to form separate mitochondrial compartments (shorter and rounder), while fusion is the merging of two or more mitochondria to form a single compartment (elongated). These two processes are under the control of the group of mitochondrial fission and fusion proteins. In the steady state, the frequencies of fusion and fission events are balanced, maintaining the overall morphology/function of the mitochondria. Changes in the balance of fission and fusion have been implicated in a number of biological processes including cell division, apoptosis, autophagy, and metabolism. Fusion is an important mechanism for the redistribution of mitochondrial proteins/genes, protecting cells from the consequences of mitochondrial DNA mutations. Increased fission and/or attenuated fusion results in mitochondrial fragmentation and dysfunction. There are limited investigations regarding the role of renal oxygenation and mitochondrial function in the kidney in various pathophysiological conditions. We will now discuss the available literature in this area in ischemia- and sepsis-associated acute kidney injury (AKI).

## Renal Oxygenation and Mitochondrial Function in AKI

### Ischemia-Reperfusion

Ischemia-reperfusion is the most common animal model of AKI studied, and it pertains to the clinical scenario of AKI seen in renal transplantation, postoperative AKI, cardiac arrest, shock, or other conditions where the RBF is interrupted or significantly diminished. The demonstration of renal tissue hypoxia during the ischemic phase is obvious, but investigators have also shown cortical and medullary hypoxia in the early reperfusion phase. This appears to be mainly due to a persistent decrease in RBF and consequently oxygen delivery, while the QO_2_ is either minimally decreased or even increased, presumably due to an increased filtered load which compels tubular Na reabsorption [8]. Using noninvasive imaging techniques such as blood oxygendependent magnetic resonance imaging (BOLD MRI), persistent renal tissue hypoxia, particularly in the outer medulla, has been demonstrated during the reperfusion phase [9, 10].

In human subjects with AKI, there are few data on the correlation between QO_2_, renal oxygen delivery, blood flow, and GFR. Therefore, the current views on renal oxygenation are largely based on experimental animal models. However, Ricksten et al. [11] have performed a number of clinical studies to characterize renal oxygenation in postoperative AKI and recently summarized their data in an excellent review. Specifically, in human subjects with a normal baseline renal function, they studied renal perfusion, filtration, and oxygenation following the development of postcardiac surgery AKI [12]. They found that, compared to the non-AKI group, in the AKI group the GFR (–57%), RBF (–40%), and TNa (–59%) were lower. Despite a reduction in the GFR, the filtered Na load, and TNa, the renal oxygen extraction (+68%) was higher and the QO_2_ factored for the GFR or TNa was increased in the AKI group. Thus, despite a decrease in tubular workload and oxygen delivery, oxygen extraction and consumption by the kidney were higher in AKI. This suggests an inefficiency of the oxygen utilized for Na transport, utilization of oxygen for other processes besides Na reabsorption, or dysfunction in the mitochondrial utilization of oxygen for oxidative metabolism.

Mitochondrial dysfunction in ischemic AKI has received significant attention in recent years. Alterations in complex I activity and mitochondrial permeability transition in isolated proximal tubular cells in hypoxiareoxygenation have been demonstrated [13]. Alterations in mitochondrial morphology and dynamics are also associated with and indicate changes in mitochondrial metabolism in pathophysiology. These processes are regulated by fission (DRP1 and Fis1) and fusion (Mfn1, Mfn2, and OPA1) proteins [14]. Brooks et al. [15] demonstrated an increased activation of Drp1 leading to mitochondrial fragmentation/fission and tubular cell apoptosis as early as 2 h after azide-induced ATP depletion in cultured rat proximal tubular cells and in vivo in ischemia-reperfusion injury. Treatment with Mdivi, a pharmacological inhibitor of Drp1, significantly ameliorated this. In human renal allografts, mitochondrial membrane permeability and the release of cyto-chrome c appear to be associated with tubular cell apoptosis [16]. Funk and Schnellmann [17] illustrated the important role of mitochondrial biogenesis in the pathogenesis of ischemic AKI by showing improved mitochondrial function and less tubular injury with pharmacological targets for the enhancement of mitochondrial biogenesis. Lastly, changes in mitochondrial structure and function have also been reproduced in vivo in mouse and rat kidneys with the use of multiphoton imaging in ischemic AKI [18].

### Sepsis-Induced AKI

While systemic hemodynamics in human sepsis are well described, the behavior of renal hemodynamics remains controversial. Until recently, renal vasoconstriction and hypoperfusion leading to ischemic injury was implicated as the key pathogenic factor in sepsis-induced AKI [19]. However, the data supporting this archetype was mainly derived from short-term animal studies using lipopolysaccharide endotoxin infusion characterized by a hypodynamic state. Recent experimental and clinical studies have challenged the paradigm of renal vasoconstriction as a prerequisite for sepsis-induced AKI by demonstrating low GFR with sustained or increased RBF [20, 21]. This, however, does not rule out alterations in the microcirculation which may still be important in the pathogenesis of sepsis-associated AKI. The role of renal oxygenation and oxygen consumption was specifically examined by Heemskerk et al. [22] in rats 3 h after induction of bacteremia or endotoxemia. At that early stage, they found a decrease in oxygen delivery either due to a decreased arterial oxygen content or a decreased renal plasma flow, but higher renal oxygen extraction and QO_2_ factored for TNa, in both bacteremic and endotoxemic rats compared to controls. Both experimental and clinical studies have demonstrated that the integrity of the tubular reabsorptive function is preserved disproportionately to the overall reduction in GFR [20, 23], but the metabolic price at which tubular reabsorption occurs appears to be increased in sepsis.

The histopathology of sepsis is characterized by a paucity of cellular death which is also disproportionate to the severity of GFR reduction [24]. This has perpetuated an interest in examining ultrastructural changes in tubular epithelium including mitochondrial morphology and function. Utilizing mouse models of sepsis (lipopolysaccharide and cecal ligation and puncture), Tran et al. [25] showed that while proximal tubular cells displayed minimal vacuolization on light microscopy, ultrastructural imaging demonstrated swollen mitochondria and rarefied cristae in proximal tubular cells. They also demonstrated suppression of PPAR-γ coactivator-1α (PGC-1α), a major regulator of mitochondrial biogenesis and metabolism.

## Conclusion

Tubular transport and oxidative metabolism are closely coupled in the kidney and are the principal determinants of intrarenal oxygenation. While there are a few investigations characterizing renal oxygenation and mitochondrial function independently in ischemic and sepsis-associated AKI, the coupling of transport and metabolism in pathophysiology has not been examined. This is a potentially fruitful area of research that should become increasingly active given the emerging data linking renal oxygenation and hypoxia to acute and chronic dysfunction in the kidney.
